# Survival After Contralateral Axillary Metastasis in Breast Cancer

**DOI:** 10.1245/s10434-024-15370-1

**Published:** 2024-05-02

**Authors:** Ji-Jung Jung, Jong-Ho Cheun, Eunhye Kang, Ikbeom Shin, Jinyoung Byeon, Hwajeong Lee, Hong-Kyu Kim, Han-Byoel Lee, Wonshik Han, Hyeong-Gon Moon

**Affiliations:** 1 Department of surgery, Seoul National University Hospital, Seoul National University College of Medicine, Seoul, Republic of Korea; 2 Department of Surgery, Seoul Metropolitan Government Seoul National University Boramae Medical Center, Seoul, Republic of Korea; 3 Cancer Research Institute, Seoul National University Hospital, Seoul, Republic of Korea

**Keywords:** Contralateral axillary metastasis, Breast cancer, Breast cancer surveillance, Locoregional recurrence, Distant metastasis

## Abstract

**Background:**

Despite stage IV categorization, survival outcomes for breast cancer patients who experience contralateral axillary lymph node metastasis (CAM) remain uncertain. This study aimed to investigate the clinical outcomes for patients with metachronous CAM to provide insights into its prognosis and treatment recommendations.

**Methods:**

This study retrospectively reviewed medical records of patients who underwent curative surgery for breast cancer and experienced CAM as the first site of distant metastasis (DM) during the follow-up period between January 2001 and April 2023. Survival outcomes of the CAM patients were compared with those of breast cancer patients with other DM via propensity score-matching (PSM).

**Results:**

The study identified 40 breast cancer patients with metachronous CAM. The estimated 5-year overall survival (OS) was 39.6%, and the progression-free survival was 39.4%. The patients with CAM exhibited marginally better OS than the patients with DM (*p* = 0.071), but survival similar to that of the patients with isolated supraclavicular node recurrence (SCN) (*p* = 0.509). Moreover, matching of CAM with DM using two PSM models showed a consistently insignificant survival difference (hazard ratio [HR], 1.47; *p* = 0.124 vs. HR, 1.19; *p* = 0.542). Ipsilateral breast tumor recurrences (IBTRs) were experienced by 12 patients before or concurrently with the CAM. These patients exhibited significantly better survival than the remaining patients (HR, 0.28; *p* = 0.024).

**Conclusion:**

The breast cancer patients with CAM showed survival similar to that for the patients with DM, supporting the current stage IV classification of the CAM. However, CAM associated with IBTR exhibited superior survival outcomes, suggesting that this subset of CAM may benefit from treatments with curative intent.

**Supplementary Information:**

The online version contains supplementary material available at 10.1245/s10434-024-15370-1.

Contralateral axillary lymph node metastasis (CAM) refers to the spread of cancer cells from a primary breast tumor to the contralateral axillary lymph node. The exact prevalence varies depending on the literature and definition, but the incidence of metachronous CAM or CAM diagnosed during surveillance after initial treatment is as low as 0.8–1.0%.^1,2^ The current guideline classifies CAM as stage IV distant metastasis (DM), anticipating incurable disease with a poor prognosis.

Despite this, recent studies have shown a better prognosis for CAM than for other stage IV diseases and have suggested re-classification of CAM as a locoregional event.^3–5^ Moreover, a large proportion of patients with CAM but no other metastasis are being treated with curative intent.^6^ According to the largest systematic review with 24 case reports, surgical treatment for CAM was administered to 38 patients, and 92% received surgical excision, with the majority (97%) undergoing an axillary lymph node dissection (ALND).^4^

Although most metachronous CAMs present as an isolated site of recurrence, a remarkable proportion (30%) arise concurrently with ipsilateral breast tumor recurrence (IBTR) or followed by IBTR.^5^ Furthermore, another study highlighted the difference in time to the development of CAM between isolated CAM (34 months) and CAM accompanied by synchronous IBTR (108–138 months).^4^ These findings suggest that distinct entities with separate prognoses may exist among patients with a diagnosis of metachronous CAM. However, to date, only one single-institution series with 47 patients investigated this and documented a statistically insignificant but slightly lower 5-year overall survival (OS) for patients with CAM accompanied by synchronous IBTR (61%) than for those without IBTR (77%).^5^

Because of the low incidence and few studies available, many controversial and unanswered questions exist regarding CAM. Moreover, its status as a highly heterogeneous disease makes it harder to predict prognosis, aligning with the findings of previous studies of metastatic breast cancer, which show wide variation in patient outcomes depending on clinicopathologic characteristics and patterns of recurrence.^7–9^ On the basis of unclear prognosis and heterogeneity in CAM, we conducted a comprehensive investigation of patients with metachronous CAM to add insight to this rarely described type of recurrence.

## Methods

### Study Design

The study included patients who underwent curative surgery for breast cancer and experienced CAM as the first site of DM during surveillance between January 2001 and April 2023 at Seoul National University Hospital. Patients who had IBTR or locoregional recurrence were included. However, the study excluded patients with a history of contralateral breast cancer or DM of another site before diagnosis of CAM.

For the patients who met the inclusion criteria, imaging tests including breast magnetic resonance imaging (MRI), chest computed tomography (CT), bone scan, and abdomen ultrasonography (USG), were checked to exclude primary cancer in the contralateral breast or recurrence at another metastatic site. This study was approved by the institutional review board of Seoul National University Hospital, and the requirement for informed consent was waived because it was a retrospective study that had no potential harm to the included patients.

### Patient Characteristics

Baseline clinicopathologic data were obtained from the comprehensive database and electronic medical record of our institution. The initial clinical and pathologic tumor-node-metastasis (TNM) stage was classified according to the eighth American Joint Committee on Cancer staging criteria.^10^ Hormone receptor (HR) status, including estrogen and progesterone receptors, was assessed by immunohistochemistry (IHC) and defined as positive when dyed more than 1%. Human epidermal growth factor receptor type 2 (HER2) status was evaluated using anti-HER2 antibodies and fluorescence *in situ* hybridization when needed. The study defined HER2 positivity as an IHC score of 3+ or gene amplification by FISH, and Ki-67 of more than 10% was defined as high according to a previous study conducted in our institution.^11^

### Surveillance and Follow-Up Evaluation

The patients who underwent surgery at our institution were regularly followed up every 6–12 months for the first 5 years, then annually up to 10 years to receive breast USG or MRI. Despite current guidelines against regular tests to detect DM, all the patients received all or most of the imaging tests including bone scans, chest CT, abdomen USG, and abdomen CT.

All the patients with isolated CAM were retrospectively reviewed for recurrence events, death, or both during surveillance. Recurrence events were classified as IBTR, regional recurrence (RR), or subsequent DM. This study defined IBTR as recurrence in the ipsilateral breast among patients who received breast-conserving surgery. Regional recurrence was defined as any recurrence in the regional lymph nodes (ipsilateral axillary, internal mammary, supraclavicular, or infraclavicular lymph nodes), ipsilateral chest wall, or skin of the breast. Accordingly, all the patients were analyzed for RR. Recurrence at any distant site except the contralateral axilla was defined as DM. Survival data were retrieved from the electric medical record and complemented with information from the population registers at the Ministry of the Interior and Safety, which use a personal identification number assigned to all Korean residents.

### Statistical Analysis

The follow-up period for OS and progression-free survival (PFS) was calculated from the date of CAM diagnosis. The Kaplan–Meier method was used to calculate the survival rates. Log-rank tests were used for comparison, and statistical significance was set at a *p* value lower than 0.05. To minimize potential selection bias between the two groups, we performed 1:4 propensity score-matching (PSM) using clinicopathologic variables shown repeatedly to be associated with prognosis after metastasis, namely, age at diagnosis of metastatic breast cancer, metastatic-free interval, HR status, HER2 status, number of metastatic organ systems involved, and presence of lymph node metastases at the time of initial breast cancer treatment.^8,12–15^

Additional PSM was performed, with prognostic factors determining the post-metastasis OS (tumor stage, HR status, Ki-67 expression level, metastatic-free interval, site of metastasis, and presence of symptom), as reported in a previous study conducted at our institution.^16^ The site of metastasis was categorized as favorable (lymph node, lung, bone) versus poor (liver, brain, multiple sites), whereas the presence of symptoms was omitted due to unavailability. All analyses were performed using R software version 3.6.3 (The R Foundation for Statistical Computing, Vienna, Austria).

## Results

### Patient Characteristics

The selection criteria for analysis were met by 40 patients. The baseline characteristics at the time of the initial diagnosis and the information on adjuvant treatments are presented in Table 1. The median age at the initial operation was 48 years (interquartile range [IQR], 39–60 years). At the initial operation 22 patients (55%) received mastectomy, and 28 patients (70%) received ipsilateral axillary lymph node dissection. Among the 28 node-positive patients, 3 patients underwent sentinel lymph node biopsy (SLNB) alone. A majority of the patients received cytotoxic chemotherapy (*n* = 36, 90%) and adjuvant radiotherapy (*n* = 27, 67.5%).

**Table 1:** Clinical characteristics of patients

Characteristic	All (*n* = 40), *n* (%)
Median age at operation: years (IQR)	48 (39–60)
Breast operation	
Breast-conserving	17 (42.5)
Mastectomy	22 (55.0)
Not performed	1 (2.5)
Axillary operation	
SLNB	11 (27.5)
ALND	28 (70.0)
Not performed	1 (2.5)
T stage^a^	
Tis	3 (7.5)
T1	8 (20.0)
T2	18 (45.0)
T3–4	10 (25.0)
Unknown	1 (2.5)
N stage^a^	
N0	11 (27.5)
N1	10 (25.0)
N2	7 (17.5)
N3	11 (27.5)
Unknown	1 (2.5)
Lymphovascular invasion	
Present	19 (47.5)
Absent	15 (37.5)
Unknown	6 (15.0)
Ki-67 index (%)	
< 10	19 (47.5)
≥ 10	20 (50.0)
Unknown	1 (2.5)
Histologic grade	
I–II	13 (32.5)
III	21 (52.5)
Unknown	6 (15.0)
Breast cancer subtype	
HR+/HER2–	12 (30.0)
HR+/HER2+	3 (7.5)
HR–/HER2+	11 (27.5)
TNBC	13 (32.5)
Unknown	1 (2.5)
Neoadjuvant chemotherapy	
Administered	19 (47.5)
Not administered	21 (52.5)
Adjuvant chemotherapy	
Administered	29 (72.5)
Not administered	11 (27.5)
Adjuvant radiotherapy	
Administered	27 (67.5)
Not administered	13 (32.5)
Adjuvant endocrine therapy	
Administered	15 (37.5)
Not administered	25 (62.5)
HER2-targeted treatment	
Administered	7 (17.5)
Not administered	33 (82.5)

IQR, interquartile range; SLNB, sentinel lymph node biopsy; ALND, axillary lymph node dissection; HR, hormone receptor; HER2, human epidermal growth factor receptor-2; TNBC, triple-negative breast cancer

^a^Stratified according to the American Joint Committee on Cancer (AJCC) 8th TNM stage, patients who underwent neoadjuvant chemotherapy were evaluated with clinical stage

The median time between the initial diagnosis and the development of CAM was 33.5 months (IQR, 19.0–74.5 months), and CAM occurred cumulatively during the observation period (Fig. S1). In most cases (*n* = 38, 95%), the diagnosis of CAM was made by pathologic or cytologic confirmation of the metastatic cancer cells in the lymph nodes. The information on the treatment of the patients after they experienced CAM are summarized in the Table 2.

**Table 2:** Treatment after the development of contralateral axillary metastasis

	All (*n* = 40), *n* (%)
Systemic therapy	38 (95.0)
Chemotherapy	29 (72.5)
Chemotherapy and endocrine therapy	7 (17.5)
Endocrine therapy	2 (5.0)
Surgical resection	25 (62.5)
ALND	23 (57.5)
Targeted nodal removal	2 (5.0)
Radiotherapy	11 (27.5)

ALND, axillary lymph node dissection

### Treatment and Survival After the Development of CAM

Most of the patients (*n* = 38, 95%) received systemic therapy after the diagnosis of CAM. Two patients received only endocrine therapy, but 36 patients (90%) received cytotoxic chemotherapy. Only one patient received axillary dissection for CAM without additional systemic treatment.

For diagnostic or therapeutic purposes, 25 patients (62.5%) underwent surgical resection of the metastatic nodes. In particular, 23 patients underwent ALND, and two patients received targeted nodal removal. Univariate analysis showed that surgical resection provided a significant survival benefit, whereas chemotherapy, radiotherapy, and hormonal treatment did not (Table S1 and Fig. S2). Multivariate analysis also suggested a statistically significant benefit associated with the surgical resection for CAM (Table S2).

**Fig. 1:**
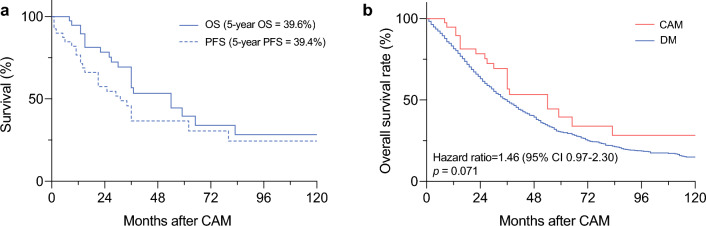
Survival analysis of patients with contralateral axillary lymph node metastasis (CAM). **a** Overall survival (OS) and progression-free survival (PFS) analyses of patients with CAM. **b** OS analysis of patients with CAM versus patients with distant metastasis (DM)

The median follow-up time from diagnosis of CAM was 36 months (IQR, 16–54 months). The 5-year OS was 39.6% and the 5-year PFS was 39.4% (Fig. 1a). An additional DM developed in 20 patients (50%), and 21 patients (52.5%) died during the follow-up period. Of the patients with disease progression, 14 (67%) presented metastases at multiple sites.

### Survival Outcome of CAM, Supraclavicular Node Recurrence, and Distant Metastasis

To determine the prognostic significance of the CAM development, we compared the survival outcome for the CAM patients with that for 40 patients who experienced supraclavicular node recurrence (SCN) and 1148 patients with DM. As shown in the Fig. 1b, the patients with CAM exhibited a marginally better OS than the patients with DM (*p* = 0.071) but an OS similar to that for the patients with isolated SCN recurrence (*p* = 0.509; Fig S3).

**Fig. 2:**
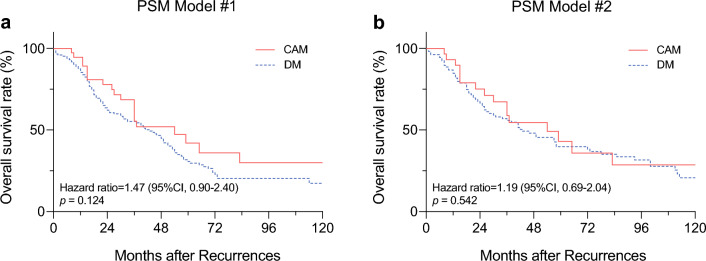
Propensity score-matched (PSM) survival analysis. **a** PSM analysis using clinicopathologic variables associated with prognosis (PSM model #1). **b** PSM analysis using clinical features at the time of metastasis (PSM model #2)

The patients with DM showed clinicopathologic features that differed significantly from those of the CAM patients. To minimize the bias, we performed a 1:4 PSM analysis to compare the survival outcomes of CAM and DM, as shown in the Table 3. After PSM, OS did not differ significantly between 150 DM patients and 39 CAM patients (Fig. 2a). Because the clinical information at the time of metastasis is also prognostic, we developed another PSM model with 110 DM patients and 31 CAM patients. However, the second PSM model also demonstrated a lack of survival difference between the CAM and DM groups (Fig. 2b).

**Table 3:** Characteristics of patients with and without adjustment for propensity score (PS) used in survival analyses

Characteristic		Overall			PS-matching			
		CAM (*n* = 39) *n* (%)	DM (*n* = 1059) *n* (%)	SMD	CAM (*n* = 39) *n* (%)		DM (*n* = 150) *n* (%)	SMD
Age at metastasis (years)	Median (IQR)	48.0 (39.0–62.5)	55.0 (45.0–63.0)	0.24	48.0 (39.0–62.5)		52.0 (44.0–60.0)	0.12
Metastatic-free interval	Median (IQR)	33.0 (18.0–72.0)	34.0 (17.0–66.0)	0.02	33.0 (18.0–72.0)		30.0 (13.0–66.0)	0.05
Hormone receptor status	Positive	15 (38.5)	695 (65.6)	0.56	15 (38.5)		55 (36.7)	0.07
	Negative	24 (61.5)	364 (34.4)	0.56	24 (61.5)		95 (63.3)	0.07
HER2 receptor status	Positive	14 (35.9)	260 (24.6)	0.24	14 (35.9)		51 (34.0)	< 0.01
	Negative	25 (64.1)	799 (75.4)	0.24	25 (64.1)		99 (66.0)	< 0.01
No. of metastatic organ systems involved	Single	39 (100.0)	830 (78.4)	0.53	39 (100.0)		150 (100.0)	0.00
	Multiple	0 (0.0)	229 (21.6)	0.53	0 (0.0)		0 (0.0)	0.00
N stage (primary cancer)	N0	11 (28.2)	284 (26.8)	0.03	11 (28.2)		41 (27.3)	< 0.01
	N1	10 (25.6)	376 (35.5)	0.23	10 (25.6)		41 (27.3)	0.02
	N2	7 (17.9)	244 (23.0)	0.13	7 (17.9)		25 (16.7)	0.05
	N3	11 (28.2)	153 (14.4)	0.31	11 (28.2)		43 (28.7)	0.02
	Unknown	0 (0.0)	2 (0.2)	0.04	0 (0.0)		0 (0.0)	0.00

CAM, Contralateral axillary lymph node metastasis; DM, distant metastasis; SMD, standardized mean difference; IQR, interquartile range; HER2, human epidermal growth factor receptor-2

### Impact of Local Recurrence Patterns on Survival Outcomes

**Fig. 3:**
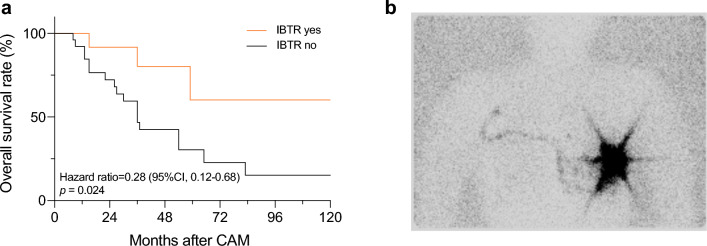
**a** 10-Year overall survival of patients with contralateral axillary lymph node metastasis (CAM) in association with ipsilateral breast tumor recurrence (IBTR). **b** Breast lymphoscintigraphy showing aberrant lymphatic drainage to contralateral axilla

Among the 40 patients who experienced CAM, 12 had IBTR before or at the time of the CAM diagnosis. These 12 patients showed a significantly better OS than the remaining 28 patients (Fig. 3a). In contrast, the patients who experienced regional recurrences (e.g., skin, chest wall) did not show such significant differences of survival.

To address the cause for the improved survival of the CAM patients with IBTR, we performed an additional analysis focusing on patients who experienced IBTR concurrently with CAM. Our separate analysis showed that these 8 patients had a better OS than the other 32 patients who presented with isolated CAM (5-year OS: 55.6% vs. 32.5%; *p* = 0.064; Fig S4). Moreover, we analyzed the lymphoscintigraphy of IBTR cases in an independent cohort of 16 patients. One of these patients (6.2%) showed lymphatic drainage to the contralateral axilla (Fig. 3b).

## Discussion

Contralateral axillary lymph node metastasis is a rare disease currently included in the stage IV category. Unlike other stage IV diseases, the majority of patients with CAM are treated with curative intent in the view that the prognosis and treatment outcome of CAM is similar to that of SCN metastasis.^3,5,6^ However, our single-center, retrospective analyses of 40 patients demonstrated a poorer prognosis for patients with CAM compared with the results of recently published case series. During a median follow-up period of 36 months, the estimated 5-year OS and PFS for the patients who experienced CAM during surveillance were respectively 39.6% and 39.4%. Compared with the patients who experienced SCN recurrence or distant organ metastasis, the patients with CAM appeared have an OS similar to that of the SCN patients, but propensity score-matched analyses demonstrated no survival difference between the CAM patients and the matched DM patients. However, subgroup analyses of those who experienced locoregional recurrence indicated a better survival rate for the patients who experienced IBTR before or at the time of CAM diagnosis. As a result, our study concluded that a subpopulation with IBTR may have a better prognosis, but there is a lack of evidence that the survival of metachronous CAM patients in general is better than that of other stage IV patients.

Previous studies of CAM comprised a small number of patients and had varying outcomes. According to four studies with fewer than 30 patients each, the OS rate ranged between 33 and 71% during median follow-up periods of 24–35 months.^1,2,17,18^ More recent studies with fewer than 60 patients each showed great improvement to a 5-year OS of 67.4–72%.^3,5^ These findings prompted a question whether CAM should be considered as an extension of locoregional disease rather than as stage IV distant metastasis. However, reclassification of CAM to N3 should be undertaken only when consistent evidence shows that the survival outcome of CAM patients surpasses that of patients with a diagnosis of stage IV distant organ metastasis. To this end, we narrowed our focus to patients who experienced CAM during the surveillance period to perform analysis among a more homogeneous population. Furthermore, to account for heterogeneity in prognosis among stage IV patients, for propensity score-matching, we used clinicopathologic variables that findings have shown repeatedly to be associated with a post-metastasis prognosis.^7,8^ Nevertheless, the results consistently showed no difference in survival between the metachronous CAM patients and the DM patients.

Likewise, treatment for CAM not accompanied by other distant-site metastasis also is controversial. Unlike patients with other metastatic diseases, many patients with isolated CAM are being treated with curative intent.^4,5^ A previous systematic review showed that up to 97.3% of patients underwent locoregional treatment including axillary surgery, radiotherapy, or both.^4^

A recent study with 60 patients demonstrated that axillary surgery significantly improved prognosis, whereas radiotherapy did not add survival benefit.^3^ Similarly, our study showed that 62.5% of the patients with CAM received surgical resection, which was associated with a significant survival benefit. Although the results of the multivariate analysis also showed significant benefit associated with surgical resection of CAM, it still is important to note that some unadjusted bias may exist in the decision to perform axillary surgery because it might have been performed for patients deemed to benefit or for selected patients who had to undergo ipsilateral breast surgery for IBTR.

Prior studies have highlighted that CAM often occurs simultaneously with or preceded by IBTR or RR, and that the presence of locoregional recurrence seemed to influence prognosis.^4^ However, existing research on this topic has been limited, with only one study to date that reported worse OS and DFS for patients with IBTR.^5^ In contrast, our findings suggest that patients with CAM and IBTR experienced improved survival. To address the reason for this improved survival, we investigated the possibility of aberrant lymphatic drainage to the contralateral axilla in IBTR cases.^19^ Unfortunately, none of the IBTR patients included in this study underwent lymphatic mapping before surgical resection of the recurrent tumor. However, in a separate group of 16 IBTR patients who underwent lymphoscintigraphy, aberrant lymphatic drainage to the contralateral axilla was observed in one case. According to previous reports regarding lymphatic scan and aberrant drainage in recurrent non-metastatic breast cancer cases, aberrant drainage was observed in 19.8–54.1%, and contralateral axillary drainage was as common as 33.3–52.2%.^20–22^ Unfortunately, even in these cases with available lymphoscintigraphy, it remains unclear whether CAM is directly related to IBTR, meaning synchronous CAM from a recurred tumor, or represents a metachronous distant metastasis originating from the previously treated primary cancer. Nonetheless, when CAM patients exhibit concurrent aberrant lymphatic drainage, it is important to consider the potential coexistence of both phenomena. In our study, 8 of the 12 patients who experienced IBTR had a simultaneous diagnosis of CAM. Although we classified these patients as metachronous CAM cases in our analysis, it is worth noting that they could potentially represent a mix of synchronous CAM from recurrent tumors and metachronous CAM from the primary tumor.

In conclusion, our study demonstrated no significant differences in survival outcome between CAM patients and those with DM. Reclassification of CAM as a locoregional disease remains challenging due to the lack of consistent evidence supporting superior outcomes. However, subgroup analysis indicated that CAM associated with IBTR had superior survival outcomes, suggesting that this subset of CAM patients may benefit from treatments with curative intent.

### Supplementary Information

Below is the link to the electronic supplementary material.

Supplementary file1 (DOCX 152 kb)
