# Supplementary material for: Survival After Contralateral Axillary Metastasis in Breast Cancer
Source: Ann Surg Oncol. 2024 May 2;31(8):5189–96. doi: 10.1245/s10434-024-15370-1 (PMC11236886; doi:10.1245/s10434-024-15370-1)
Supplement: Supplementary file 1 — Supplementary file1 (DOCX 152 kb) [file 10434_2024_15370_MOESM1_ESM.docx]

**eSupplemental material**

**Survival after the Contralateral Axillary Metastasis in Breast Cancer**

**Table of contents**

Cumulative incidence of contralateral axillary lymph node metastasis after primary tumor Fig. S1

Log-rank analyses for overall survival based on treatment modality for contralateral axillary lymph node metastasis Table. S1

Survival analysis by treatment methods for contralateral axillary lymph node metastasis Fig. S2

Univariate and multivariate analyses for overall survival of patients with contralateral axillary lymph node metastasis Table. S2

Survival analysis for patients with contralateral axillary lymph node metastasis compared to patients with recurred isolated supraclavicular node metastasis Fig. S3

Survival analysis by concurrent diagnosis of ipsilateral breast tumor recurrence Fig. S4

**Figure S1. Cumulative incidence of contralateral axillary lymph node metastasis after primary tumor**

**Table S1. Log-rank analyses for overall survival based on treatment modality for contralateral axillary lymph node metastasis**

| Variables | N | Log-rank analysis | |
| --- | --- | --- | --- |
|  |  | Hazard ratio [95% CI] | *p*-value |
| Axillary surgery | 25 | 0.40 (0.15-0.85) | 0.023 |
| Chemotherapy | 36 | 2.81 (0.76-10.3) | 0.271 |
| Radiotherapy | 11 | 1.03 (0.41-2.56) | 0.946 |
| Hormonal treatment | 9 | 0.52 (0.21-1.32) | 0.217 |

**Figure S2. Survival analysis by treatment methods for contralateral axillary lymph node metastasis**


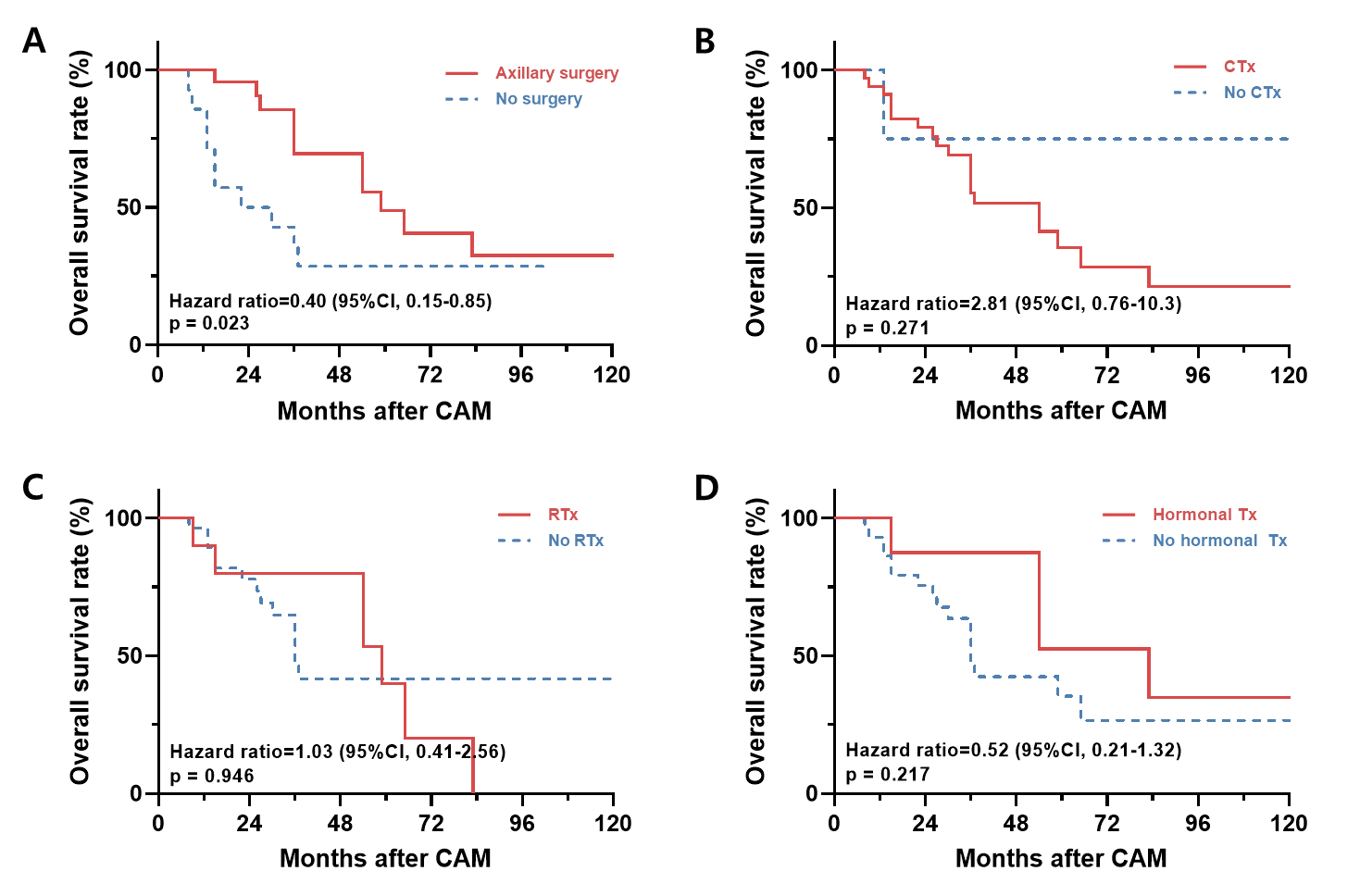


**Table S2. Univariate and multivariate analyses for overall survival of patients with contralateral axillary lymph node metastasis**

| Variable | **Univariate** | | **Multivariate** | |
| --- | --- | --- | --- | --- |
|  | **HR (95% CI)***^1^* | **P value** | **HR (95% CI)***^1^* | **P value** |
| Age at operation | 1.00 [0.97, 1.04] | 0.942 |  |  |
| Breast operation |  |  |  |  |
| BCS | — |  | — |  |
| Mastectomy | 5.40 [1.81, 16.1] | **0.003** | 8.15 [0.84, 79.3] | 0.071 |
| Axillary operation |  |  |  |  |
| SLNB | — |  |  |  |
| ALND | 2.37 [0.69, 8.10] | 0.169 |  |  |
| T stage |  |  |  |  |
| Tis-T1 | — |  | — |  |
| T2 | 2.24 [0.62, 8.15] | 0.221 | 0.63 [0.12, 3.36] | 0.587 |
| T3-4 | 4.03 [1.03, 15.7] | **0.045** | 0.29 [0.04, 2.28] | 0.237 |
| N stage |  |  |  |  |
| N0 | — |  | — |  |
| N1 | 2.27 [0.46, 11.3] | 0.317 | 7.57 [0.39, 145] | 0.179 |
| N2 | 5.77 [1.01, 33.0] | **0.049** | 4.49 [0.22, 91.7] | 0.329 |
| N3 | 7.43 [1.56, 35.3] | **0.012** | 7.12 [0.33, 155] | 0.212 |
| Lymphovascular invasion |  |  |  |  |
| Absent | — |  |  |  |
| Present | 1.98 [0.70, 5.64] | 0.2 |  |  |
| Ki-67 index |  |  |  |  |
| < 10% | — |  |  |  |
| ≧ 10% | 1.54 [0.63, 3.76] | 0.347 |  |  |
| Histologic grade |  |  |  |  |
| I-II | — |  |  |  |
| III | 1.67 [0.58, 4.80] | 0.339 |  |  |
| Breast cancer subtype |  |  |  |  |
| HR+/HER2− | — |  |  |  |
| HR+/HER2+ | 1.10 [0.22, 5.47] | 0.91 |  |  |
| HR−/HER2+ | 1.11 [0.31, 4.02] | 0.869 |  |  |
| TNBC | 2.29 [0.78, 6.74] | 0.131 |  |  |
| Treatment for primary tumor |  |  |  |  |
| Neoadjuvant chemotherapy | 0.89 [0.37, 2.11] | 0.788 |  |  |
| Adjuvant chemotherapy | 1.02 [0.37, 2.81] | 0.963 |  |  |
| Adjuvant radiotherapy | 0.59 [0.23, 1.53] | 0.278 |  |  |
| Adjuvant endocrine therapy | 0.52 [0.21, 1.29] | 0.158 |  |  |
| HER2-targeted treatment | 0.52 [0.12, 2.30] | 0.391 |  |  |
| Ipsilateral breast tumor recurrence |  |  |  |  |
| No | — |  | — |  |
| Yes | 0.28 [0.12, 0.68] | **0.024** | 2.82 [0.16, 49.9] | 0.479 |
| Treatment after CAM |  |  |  |  |
| Surgical resection | 0.40 [0.15, 0.85] | **0.023** | 0.19 [0.04, 0.81] | **0.025** |
| Chemotherapy | 2.81 [0.76, 10.3] | 0.271 |  |  |
| Radiotherapy | 1.03 [0.41, 2.56] | 0.946 |  |  |
| Endocrine therapy | 0.52 [0.21, 1.32] | 0.217 |  |  |

Abbreviations: BCS, breast-conserving surgery; SLNB, sentinel lymph node biopsy; ALND, axillary lymph node dissection; HR, hormone receptor; HER2, human epidermal growth factor receptor-2; TNBC, triple negative breast cancer; CAM, contralateral axillary lymph node metastasis

**Figure S3. Survival analysis for patients with contralateral axillary lymph node metastasis compared to patients with recurred isolated supraclavicular node metastasis**

**Figure S4. Survival analysis by concurrent diagnosis of ipsilateral breast tumor recurrence**
